# Minimally Invasive Technique for PMMA Augmentation of Fenestrated Screws

**DOI:** 10.1155/2015/979186

**Published:** 2015-05-14

**Authors:** Jan-Helge Klingler, Christoph Scholz, Evangelos Kogias, Ronen Sircar, Marie T. Krüger, Florian Volz, Christian Scheiwe, Ulrich Hubbe

**Affiliations:** Department of Neurosurgery, Freiburg University Medical Center, Breisacher Straße 64, 79106 Freiburg im Breisgau, Germany

## Abstract

*Purpose*. To describe the minimally invasive technique for cement augmentation of cannulated and fenestrated screws using an injection cannula as well as to report its safety and efficacy. *Methods*. A total of 157 cannulated and fenestrated pedicle screws had been cement-augmented during minimally invasive posterior screw-rod spondylodesis in 35 patients from January to December 2012. Retrospective evaluation of cement extravasation and screw loosening was carried out in postoperative plain radiographs and thin-sliced triplanar computed tomography scans. *Results*. Twenty-seven, largely prevertebral cement extravasations were detected in 157 screws (17.2%). None of the cement extravasations was causing a clinical sequela like a new neurological deficit. One screw loosening was noted (0.6%) after a mean follow-up of 12.8 months. We observed no cementation-associated complication like pulmonary embolism or hemodynamic insufficiency. *Conclusions*. The presented minimally invasive cement augmentation technique using an injection cannula facilitates convenient and safe cement delivery through polyaxial cannulated and fenestrated screws during minimally invasive screw-rod spondylodesis. Nevertheless, the optimal injection technique and design of fenestrated screws have yet to be identified. This trial is registered with German Clinical Trials DRKS00006726.

## 1. Introduction

Pedicle screw-rod instrumentation is an accepted technique to achieve rigid internal fixation in degenerative, deformative, tumor, and trauma disorders of the spine [1, 2]. With an aging patient population, spine surgeons encounter the challenge to obtain and maintain fixation in an osteoporotic spine [2, 3]. As the bone-screw interface is generally the region most susceptible to loosening and failure, modern techniques aim to improve the fixation of screws particularly in osteoporotic vertebras [3, 4]. Cement augmentation of screws with polymethylmethacrylate (PMMA) has been reported to increase resistance to pullout and toggle failure [3, 5–7]. However, the use of PMMA involves the risk of cement extravasation, which can lead to neural compression, neurological deficits, or pulmonary embolism [8]. Fenestrated screws have been developed to increase convenience and safety of cement delivery [9–11]. With the evolution of minimally invasive spinal fixation procedures comes the need for percutaneous cement delivery through polyaxial fenestrated screws and mounted screw extenders [12, 13]. The purpose of this study is to investigate the efficacy and safety of the minimally invasive technique for cement augmentation of cannulated, fenestrated screws using an injection cannula.

## 2. Methods

### 2.1. Patients

This study is a retrospective observational trial to assess the feasibility, effectiveness, and complication rate of injecting bone cement through cannulated and fenestrated screws in minimally invasive spine stabilization procedures. We identified 35 patients in our database of a single center who underwent minimally invasive posterior stabilization of the thoracic and lumbar spine with cement-augmented fenestrated screws due to degenerative/deformative disorders, spinal trauma, or pathological fracture between January and December 2012 (Table 1).

### 2.2. Surgical Treatment

The surgical technique of performing minimally invasive stabilization procedures with screw-rod instrumentation (CD Horizon Sextant II or CD Horizon Longitude, Medtronic, Minneapolis, USA) has been described in detail elsewhere [14]. For minimally invasive thoracic instrumentation we routinely use intraoperative spinal navigation for accurate screw placement (Cart II system, Stryker, Freiburg, Germany; software: SpineMap 3D navigation) [15, 16]. Usually, no drain was used in minimally invasive spine stabilization procedures. Patients were allowed to ambulate in the morning of the first postoperative day without orthosis, unless the patient's clinical status prohibited mobilization.

### 2.3. Cement Injection Technique

PMMA augmentation of the screws was performed at the discretion of the surgeon based on the knowledge of diagnosed osteoporosis or tactile findings during surgery. If the surgeon noticed abnormally reduced bone resistance while introducing the Jamshidi needle into the vertebral body, PMMA augmentation was performed [17, 18].

**Figure 1:**
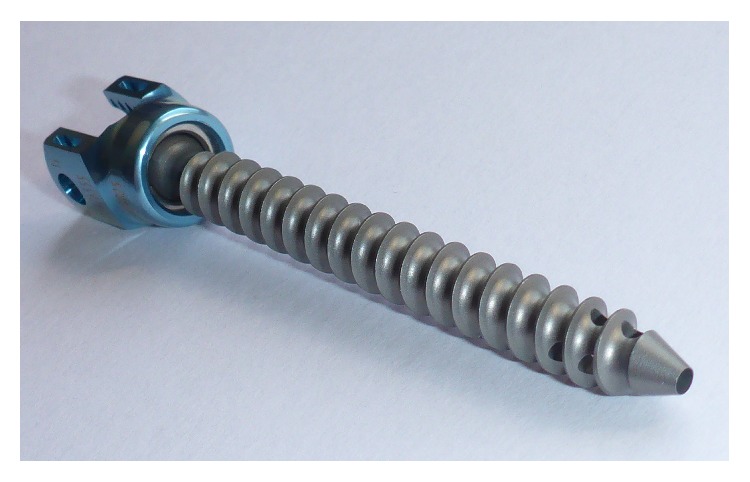
Polyaxial cannulated and fenestrated screw. The screw is fully cannulated with a total of six distal fenestrations (four fenestrations are in sight). Note the polyaxial screw head.

**Figure 2:**
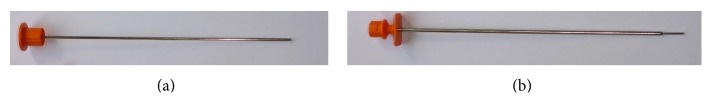
Bone cement injection cannula. The injection cannula (b) can be filled with 1.5 mL of bone cement. With the pusher (a), the bone cement is poured in the cannulated screw and the surrounding vertebral body.

**Figure 3:**
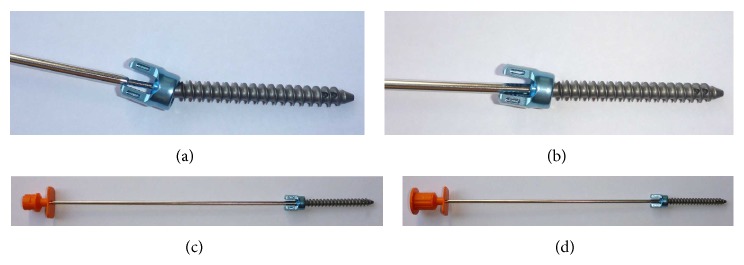
Exemplary assembly of injection cannula and polyaxial cannulated and fenestrated screw for percutaneous cement augmentation. The injection cannula is initially inserted unfilled into the polyaxial screw head (a) to check the proper fit and entry trajectory (b). After removing and filling the injection cannula with bone cement outside of the patient's body, the filled injection cannula is reinserted (c). Gradually inserting the pusher into the injection cannula (under fluoroscopic monitoring) bone cement is injected into the screw and surrounding vertebral body through the distal fenestrations (d) (bone cement not shown).

After screwing in the cannulated and fenestrated screws (CD Horizon Fenestrated Screw Spinal System, Medtronic; Figure 1), the bone cement injection cannulas (bone cement metallic injection cannula, Tsunami Medical, San Possidonio, Italy, distributed by Maxxspine, Bad Schwalbach, Germany, Figure 2) were first inserted empty into the polyaxial screw heads to check the proper fit and entry trajectory (Figure 3). After removal of injection cannulas, the PMMA cement (VertaPlex 1/2 Dose, Stryker, Duisburg, Germany) was prepared and filled into the injection cannulas, which can hold 1.5 mL of cement. The filled injection cannulas were reinserted into the screw heads sealed to avoid cement emersion into the screw heads, which could preclude rod insertion. Injection was performed with a toothpaste-like consistency of the cement. Per screw, approximately 2 mL of cement was injected in the lumbar spine and 1.5 mL of cement in the thoracic spine. For every 0.3–0.5 mL of cement injection, cement distribution was checked with fluoroscopic images in lateral projection. In case of evidence of epidural, intradiscal, or prevertebral/intravenous cement extravasation, the injection of cement was stopped.

### 2.4. Radiographic and Complication Assessment

Cement extravasation was postoperatively evaluated in plain radiographs and additionally in available computed tomography (CT) scans using integrated software (IMPAX EE R20 VIII, Agfa HealthCare, Mortsel, Belgium). They were classified into prevertebral, paravertebral, epidural, and intradiscal cement extravasations. Moreover postoperative radiographic imaging was evaluated regarding screw loosening or breakage. Screw loosening was certified if radiographs or CT showed a clear zone around the screw and the radiolucency was 1 mm or wider at the bone-screw interface. Loss of lordosis from postoperative to final follow-up was calculated measuring the Cobb angle within the instrumented spine region in lateral plain radiographs. Complications and reoperations were gathered from patient records.

### 2.5. Statistical Analysis

Descriptive statistics were used to describe the basic characteristics of the data in the study. Results were expressed as means with standard deviations. Prism 6 for Mac (GraphPad Software Inc., La Jolla, USA) was used as statistical software.

## 3. Results

### 3.1. Demographics

A total of 157 cannulated and fenestrated pedicle screws had been cement-augmented in 35 patients during minimally invasive posterior screw-rod spondylodesis. Surgery was mainly performed in the lumbar spine due to degenerative/deformative disorders (Table 1). Most operations were performed as minimally invasive transforaminal lumbar interbody fusion (MIS TLIF) (24/35 patients, 68.6%). Further instrumentation techniques included minimally invasive posterior screw-rod instrumentation only (2/35 patients, 5.7%), in combination with vertebral body replacement (6/35 patients, 17.1%) or in combination with balloon kyphoplasty (3/35 patients, 8.6%). Mean follow-up time was 12.8 months.

### 3.2. Radiographic Assessment, Cement Extravasations

Cement-augmented pedicle screws were largely placed in the lower lumbar spine.  Table 2 demonstrates the distribution of minimally invasively cement-augmented screws and frequencies of cement extravasations assigned to the level of screw implantation. Overall, 27/157 (17.2%) cement extravasations were detected, at which multiple cement extravasations of one single level had been included. Most cement extravasations were located prevertebrally (20/27, 74.1%) and paravertebrally (4/27, 14.8%) (Table 3). These cement extravasations were often identified in pre- and paravertebral veins and were altogether small in amount (Figure 5). Two intradiscal (7.4%) and one minor epidural (3.7%) cement extravasations were discovered (Figure 5). None of the cement extravasations was causing a clinical sequela like a new neurological deficit.

Loss of lordosis during available follow-up time for plain radiographs (7.9 months) was 1.6° ± 3.7°.

### 3.3. Complications

We observed no mortality or cementation-associated complications like pulmonary embolism or hemodynamic insufficiency.

Screw loosening was found in one patient with minimally invasive posterior screw-rod spondylodesis L2–L4 in combination with lateral vertebral body replacement of L3 due to osteoporotic compression fracture of L3 13 months postoperatively at scheduled follow-up. Although bony fusion has not yet been achieved, revision surgery was not performed since the patient did not complain about a relevant pain level. Further follow-up examinations have been scheduled to assess fusion status and to avoid missing an early kyphotic deformity.

One patient experienced a new slight paresis of the left foot elevator (grade 4 according to the British Medical Council) after MIS TLIF. Since postoperative CT only discovered a minimal prevertebral cement extravasation, this complication was attributed to intraoperative manipulation of the L5 nerve root.

Further complications occurred, which we do not associate with cement augmentation of the screws: one superficial revision surgery 4 weeks after surgery due to wound dehiscence; one revision surgery 12 days after surgery due to epidural empyema; one revision surgery 12 days after surgery due to patient fall with screw breakage; one revision surgery 7 months after surgery due to screw breakage of a noncemented screw.

## 4. Discussion

**Figure 4:**
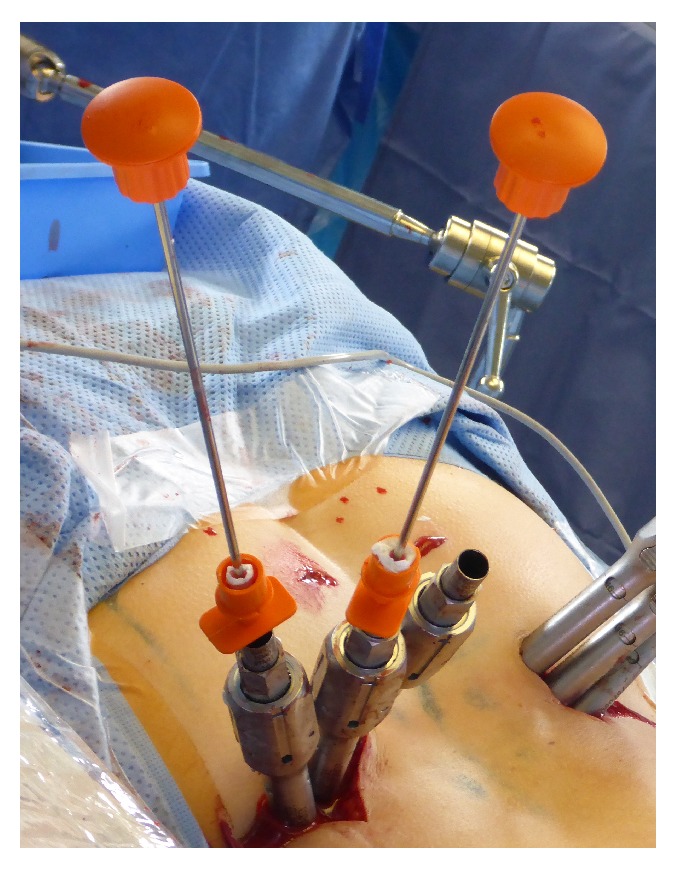
Intraoperative setting. The injection cannulas had been filled with bone cement and had been introduced through the screw extenders into the screw heads. The pushers were inserted to inject the bone cement through the cannulated screws and their fenestrations in the distal third of the thread into the vertebral body under fluoroscopic monitoring.

Performing percutaneous cement-augmentation of cannulated and fenestrated screws is a further development in minimally invasive spine surgery [12, 13]. Since the inserted polyaxial screws are mounted with screw extenders (Figure 4), a connection device has to be used for injecting bone cement. We investigated the application and results using an injection cannula in 157 minimally invasive cement-augmented screws in 35 patients. In our experience, the injection cannula warrants a proper fit of its tip in the screw head and, hence, minimizes the risk of cement extravasation in the screw head. This is important, since hardened cement in the screw head might preclude minimally invasive insertion of the rod. Accordingly, we did not experience this phenomenon in our series. A further advantage of the injection cannula is the compatibility with different spine fixation systems (e.g., CD Horizon Sextant II, CD Horizon Longitude, or CD Horizon Sextant Legacy (Medtronic, Minneapolis, USA)).

Since screw loosening only occurred in one of 157 minimally invasive cement-augmented screws (0.6%) in our series, we believe that cement-augmentation of fenestrated screws is an effective technique to support the fixation of a screw-rod spondylodesis in osteoporotic or osteopenic vertebras. Restrictively it has to be noted that the mean follow-up time of 12.8 months is rather short and fusion status was not assessed. Therefore, additional screw loosening might occur in the further course if bony fusion has not yet been achieved. Another study investigated open fusion procedures and showed no screw loosening (assessed in plain radiographs) at 12-month follow-up in 27 patients with 149 cement-augmented screws [19].

### 4.1. Other Clinical Studies Using Fenestrated Screws

Only four clinical studies have been published that examined cement augmentation using fully cannulated and fenestrated screws in spine stabilization procedures [10, 12, 13, 20]. Two of these studies used minimally invasive techniques [12, 13] with a total of 27 patients. First, Lubansu et al. [12] performed percutaneous cement augmentation of 78 fenestrated screws (titanium Expedium fenestrated screw, VIPER MIS Spine System, DePuy Spine) in 15 elderly osteoporotic patients. They used a cement delivery system (V-MAX, DePuy Spine) in combination with a specifically designed connector for percutaneous cement injection through the screw extenders. The authors evaluated cement extravasation on plain radiographs and observed 5 cement extravasations in 78 screws (6.4%) in 5 patients (33.3%), none of them classified as symptomatic. They stated two complications not associated with cement augmentation. The authors found no screw loosening after a mean follow-up of 36 months. Second, Pesenti et al. [13] performed percutaneous cement augmentation of 96 fenestrated screws (Longitude, Medtronic, or Mantis, Stryker) in 12 patients. No loosening or pullout of screws was observed in CT at the last follow-up. One cement-related pulmonary embolism occurred and was attributed to too liquid cement.

The other two studies examined cement-augmented fenestrated screws in open spine surgery. Amendola et al. [10] performed open cement augmentation of 81 monoaxial fenestrated screws (Legacy, Medtronic) in 21 patients. No loosening or pullout of screws was found in CT. The authors reported 5 cement extravasations in 81 screws (6.2%) in 5/21 patients (23.8%). One cement extravasation led to nerve root palsy; another one was noticed and removed during surgery without neurologic sequelae. The remaining three were small epidural cement extravasations stated as asymptomatic. Chang et al. [20] performed open cement augmentation in 255 monoaxial cannulated screws with one side hole (Wellong BMI Medical, Taiwan) in 45 patients. The authors evaluated 121 cement-augmented screws on CT and recorded 17 cement extravasations (14.0%) in 21 patients. One patient with epidural cement leakage had persistent left thigh pain after surgery; the remaining cement extravasations were reported to be “spotty or linear” without causing symptoms.

### 4.2. Further Techniques of Cement Augmentation

An earlier developed method is the retrograde injection technique. After preparing the screw tract by inserting and removing the screw, the bone cement is injected into the tract inside the vertebral body from anterior to posterior. Before the cement sets, the definite screw is inserted [21, 22]. Another method for screw augmentation is to perform an initial vertebroplasty or balloon kyphoplasty [19, 23]. A biomechanical study showed that balloon kyphoplasty augmentation is not superior to vertebroplasty augmentation in regard to pullout force [23]. Another technique is to coat solid screws with approximately 1 mL of PMMA cement before insertion [17]. The biomechanical effect of this technique is arguable.

The retrograde injection technique might carry an increased risk of epidural cement extravasation, since the bone cement might leak through an unrecognized violation of the pedicle wall while inserting the screw [20]. Accordingly, Chang et al. [20] reported a lower rate of cement extravasation using fenestrated screws compared to the retrograde injection method (14.0% versus 26.2% cemented screws). Using the retrograde injection method, Frankel et al. [24] stated asymptomatic cement extravasations in 9/158 screws (5.7%) in 9/23 patients (39.1%, evaluation on radiographs), and Hu et al. [11] observed asymptomatic cement extravasations in 26/145 screws (17.9%, evaluation on CT). Cement augmentation using fenestrated screws resulted in cement extravasation in up to 14% of screws in the current literature, though largely being asymptomatic [10, 12, 13, 20].

**Figure 5:**
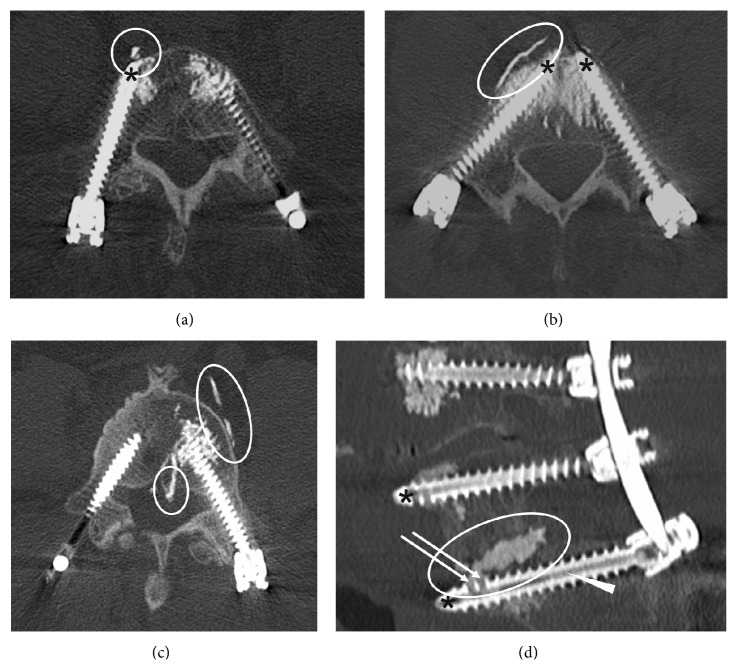
Cement extravasations. Postoperative computed tomography scans showing prevertebral ((a), (b)), paravertebral (c), epidural (c), and intradiscal (d) cement extravasations (encircled). Arrows (d) indicate fenestrations in the distal third of the thread; the arrowhead (d) indicates the hollow central shaft of the cannulated screw now filled with PMMA cement. Long screws with the tip of cannulated screws close to the anterior cortex of the vertebra (asterisks) might provoke prevertebral and paravertebral cement extravasations. Notice that the right screw (c) has not been cement-augmented due to local sclerosis of the vertebral body and therefore sufficient primary fixation strength.

In our series, we observed cement extravasations in 27/157 fenestrated screws (17.2%) in 17/35 patients (48.6%). These numbers are in the upper range of reported frequencies of cement extravasations. This may be due to recording even the smallest cement extravasations on CT in our study (Figure 5(a)). Another explanation of the relatively high number of prevertebral cement extravasations (20/27 cement extravasations, 74.1%) might be our insertion technique of screws. As can be seen in Figure 5, we tend to implant rather longer than shorter screws up to the anterior cortex of the vertebral body in order to increase the primary fixation strength. Performing cement augmentation at this position might more frequently lead to prevertebral cement extravasation through the tip of the screw.

More importantly, none of the cement extravasations in our study was causing a clinical sequela. Moreover, no pulmonary embolism, hemodynamic insufficiencies, or deaths had been observed. Therefore, all cement extravasations could be classified as asymptomatic.

However, when comparing different studies, one must take into account the imaging method used for evaluating cement extravasation. The frequency of cement extravasation is underestimated in plain radiographs compared to CT [8, 25].

### 4.3. Limitations of the Study

Obviously, the retrospective design is a methodological weakness. Furthermore, a comparison group might have helped to take the data in context with other cement augmentation techniques. The primary purpose of the study was to present the surgical technique; therefore the follow-up period is relatively short.

## 5. Conclusions

The reported minimally invasive technique with the aid of the presented injection cannula facilitates convenient and safe cement augmentation of polyaxial cannulated and fenestrated screws without increased complication rates regarding symptomatic cement extravasation or screw loosening. Nevertheless, the optimal injection technique and design of fenestrated screws have yet to be identified.

## Figures and Tables

**Table 1:** Patient characteristics. The table shows demographics, underlying cause for performing minimally invasive cement-augmented screw-rod spondylodesis and spine region of the instrumentation.

Demographics	
Number of patients	35
Patient age (y)^#^	72.8 ± 8.8
Sex (female : male)	25 : 10
Body mass index (kg/m^2^)^#^	27.3 ± 4.8
Diagnosis	
Degenerative/deformative disorder	22
Spinal trauma/osteoporotic compression/burst fracture	6
Spinal tumor/metastasis	7
Location of instrumentation	
Thoracic spine	2
Thoracolumbar junction	2
Lumbar spine	31

^#^Data are presented as mean with standard deviation.

**Table 2:** Distribution of cement extravasations. The table shows the numbers and frequencies of cement extravasations assigned to the level of minimally invasively cement-augmented pedicle screws. Note that the count of cement extravasations implies the assessment based on plain radiographs and computed tomography together.

Level of cemented screw	Count of screws studied	Count of cement extravasations	Percentage of cement extravasations	Symptomatic cement extravasations
Th1	0	n/a	n/a	n/a
Th2	2	0	0.0%	0
Th3	4	0	0.0%	0
Th4	2	0	0.0%	0
Th5	4	0	0.0%	0
Th6	2	0	0.0%	0
Th7	4	0	0.0%	0
Th8	0	n/a	n/a	n/a
Th9	2	0	0.0%	0
Th10	2	0	0.0%	0
Th11	2	1	50.0%	0
Th12	2	0	0.0%	0
L1	6	0	0.0%	0
L2	14	1	7.1%	0
L3	12	2	16.7%	0
L4	54	13	24.1%	0
L5	41	9	21.9%	0
S1	4	1	25.0%	0

Overall	157	27	17.2%	0

n/a: not applicable.

**Table 3:** Cement extravasations. The table shows numbers and locations of cement extravasations. Postoperative radiographs were available in all patients. Postoperative computed tomography (CT) was available in 24/35 patients (68.6%). Beside all cement extravasations detected on plain radiographs, CT additionally demonstrated slight prevertebral, paravertebral, and epidural cement extravasations.

Location of cement extravasation	Count on plain radiographs	Additional counts on CT
Prevertebral	18	2
Paravertebral	0	4
Epidural	0	1
Intradiscal	2	0

## Abbreviations

ap: Anterior-posterior
CT: Computed tomography
MIS TLIF: Minimally invasive transforaminal lumbar interbody fusion
PMMA: Polymethylmethacrylate.
